# Loss of epithelial markers is an early event in oral dysplasia and is observed within the safety margin of dysplastic and T1 OSCC biopsies

**DOI:** 10.1371/journal.pone.0187449

**Published:** 2017-12-07

**Authors:** Zahra Abdalla, Tanya Walsh, Nalin Thakker, Christopher M. Ward

**Affiliations:** 1 Stem Cell Research Group, Manchester Dental School, Manchester, United Kingdom; 2 Department of Histopathology, Manchester Royal Infirmary, Manchester, United Kingdom; Technische Universitat Dresden, GERMANY

## Abstract

Oral squamous cell carcinoma (OSCC) is a highly aggressive cancer that is associated with poor 5-year patient survival. Disease treatment is further compounded by the difficulty in predicting pre-cancerous tissues that will progress to OSCC and the high recurrence rates following surgical resection. Here we have assessed expression of the oral epithelial markers E-cadherin, EMP1 and 5T4 and the pro-invasive N-cadherin proteins using fully characterised antibodies and quantitative immunofluorescence microscopy in normal tissue (NT), fibroepithelial polyp (FEP), low-grade dysplasia (LGD), high-grade dysplasia (HGD), T1 OSCC and T4 OSCC biopsies. Decreased E-cadherin expression was associated with FEP, LGD and HGD biopsies, demonstrating that loss of E-cadherin is an early event within abnormal epithelium and occurs in the absence of an E- to N-cadherin switch, the latter of which was only observed in T4 OSCC. Furthermore, loss of E-cadherin and EMP1 is an indicator of LGD (p = 0.0006) and loss of E-cadherin, EMP1 and 5T4 an indicator of HGD (p = 0.0006). Expression patterns of E-cadherin, EMP1 and N-cadherin could predict abnormal epithelium in LGD, HGD, T1 and T4 OSCC biopsies (z-value = 0 for all disease grades) and allowed classification of LGD (z = 1.47), HGD (z = 2.138), T1 (z = 1.05) and T4 OSCC (z = 1.49) biopsies. Therefore, these markers provide a useful means to predict abnormal epithelium in patient biopsies. Linear regression and coefficient of determination analysis revealed positive correlation with a NT>LGD>HGD disease transition but low correlation with a putative HGD>T1 OSCC>T4 OSCC disease transition. Furthermore, expression of E-cadherin, EMP1, 5T4 and N-cadherin in pathologically normal surgical safety margins of LGD, HGD and T1 OSCC patient biopsies revealed significant differences to NT and the use of safety margins or FEP as ‘normal tissue’ controls introduced Type II errors in all patient cohorts. This work forms the basis for further investigation of the role of E-cadherin loss in abnormal epithelium and in the development of automated analyses for use in cancer diagnostics.

## Introduction

Oral squamous cell carcinoma (OSCC) is the most common cancer in the oral cavity, representing at least 90% of such malignancies, and is associated with poor 10-year survival rate compared to other cancers. Oral epithelial dysplasia is believed to be one of the progenitor populations from which OSCC arises, with transformation rates reported between 0.3 and 17.5% [1], depending on the population studied [2–5]. The mechanism by which normal oral epithelium becomes dysplastic and transforms into OSCC is not well understood and the current treatment strategy is resection of the diseased tissue to leave a disease-free margin of at least 5mm [6]. However, OSCC and dysplasia are associated with high recurrence rates at or near the original site, even in histologically and clinically normal tissue, leading to proposal of the field cancerization theory [7] and the multi-foci growth potential of cancer cells [8,9]. Some genetic and molecular alterations have been reported in the “normal mucosa” beyond the tumour resection boundaries, with mutation of the p53 gene [10–12] reported in tumour- and premalignant lesion-adjacent mucosa. Shaw et al (2013) [13] have recently reported that a significant proportion of OSCC surgical margins exhibited abnormal CpG island methylation[13], although the results were not prognostic, showing a clear need for the investigation of the epithelial phenotype within tissue associated with OSCC genesis.

Alterations in cell adhesion are required for normal embryonic development but can cause significant defects in tissue architecture and may lead to cancer development and tumour cell metastasis in established epithelium[14]. Understanding abnormalities associated with pre-cancerous lesions may allow increased knowledge of their potential transition to tumours and could lead to identification of biomarkers that can predict lesions at high risk of tumour transition and the presence of abnormal cells in histologically normal surgical margins. Much work has been established on the use of E-cadherin as a marker of dysplasia/tumour progression in OSCC, however, the conclusions from these studies are varied and investigation of expression in pre-cancerous tissues is limited. Studies have shown high levels of E-cadherin expression at the plasma membrane of normal oral epithelium and, generally, decreased expression is observed in dysplastic tissue and OSCC [15–17]. Whilst some reports suggest that E-cadherin expression is a useful biomarker of malignant transformation [18], Sridvevi et al [19] recently concluded that E-cadherin is of questionable use as a prognostic marker. These differing results likely reflect the method of analysis of protein expression and the antibody used to detect the antigen. Interestingly, many studies do not characterise the antibodies used for marker identification and many analyses use subjective methods to assess marker expression levels. Therefore, there is a requirement for additional markers for the determination of abnormal epithelial cells and the development of automated systems for the quantitative determination of their expression.

We have previously shown that inhibition of E-cadherin in embryonic stem cells (ESCs) induces a mesenchymal phenotype, increased cellular proliferation and altered global transcript expression in the absence of a characteristic epithelial-mesenchymal transition (EMT) event [20–23]. In addition, loss of E-cadherin function in ESCs also resulted in altered localisation of cell surface proteins, such as the 5T4 oncofoetal antigen, Epithelial Membrane Protein-1 (EMP1) and CD44 [20,21,23,24]. Thus, we have hypothesised that loss of E-cadherin will be an early event associated with abnormal epithelial cell growth (i.e. dysplasia), potentially leading to a growth advantage for the cells in the absence of EMT markers, such as N-cadherin [23]. In this study, we have assessed expression and localisation of E-cadherin, EMP1, 5T4, N-cadherin and CD44 in normal tissue, fibroepithelial polyp, low-grade dysplasia, high-grade dysplasia, T1 OSCC and T4 OSCC biopsies using fully characterised antibodies and immunofluorescence microscopy to determine cellular localisation and quantitative spatiotemporal expression.

## Materials and methods

### Patient biopsies

Patient biopsies were surgically excised retrospective specimens from 10 patients with fibroepithelial polyps, 20 patients with low-grade dysplasia, 16 patients with high-grade dysplasia, 5 patients with T1 OSCC and 10 patients with T4 OSCC. All biopsies were provided as 4μm sections on Apes coated slides from paraffin-embedded blocks from St. Mary’s Hospital Pathology Department, Manchester, UK. The study was reviewed and approved by The University of Manchester Ethics Committee under National Research Ethics Service (NRES) application 08/H1006/21 before the study began. Pathological staging of samples was performed by a registered pathologist in St Mary’s Hospital Pathology Department. Five samples of “normal” tongue tissue sections were purchased from AMS Biotechnology (Abingdon, UK) and seven sections from Abcam Plc (Cambridge, UK). Marker expression in surgical margins of LGD, HGD and T1 OSCC samples was performed on biopsies that exhibited normal histology and a clear surgical safety margin. Of the 20 LGD, 16 HGD and 5 T1 OSCC biopsies, a total of 9, 7 and 5 biopsies exhibited histologically normal tissue within the surgical safety margin, respectively, and were used for further study.

### Western blot analysis

1 x 10^7^ cells were lysed using Radio-Immunoprecipitation Assay (RIPA) buffer (0.1ml of 50 mM Tris, pH 8.0, 150 mM NaCl, 1% NP-40, 0.5% sodium deoxycholate and 0.1% SDS; Sigma-Aldrich, Dorset, UK) containing protease cocktail inhibitor (Roche Applied Science, Sussex, UK). Samples were incubated on ice for 30 minutes prior to centrifugation at 14,000g for 5 minutes, cell debris was discarded and cell lysate was stored at -80°C prior to use. 10μl of cell lysate was mixed 1:1 with 2x Laemmli buffer, separated by SDS-PAGE and transferred to Hybond-ECL nitrocellulose membrane (Amersham biosciences, Bucks, UK) using a Trans blot SD blotter (Biorad, Hertfordshire, UK). The membrane was incubated with appropriate primary and secondary (-HRP) antibodies diluted in blocking buffer (5% dry milk in TBS), exposed to ECL detection reagent (GE Healthcare, Bucks, UK) and visualised using autoradiography film manually developed using Kodak GBX developer and fixer solutions (Sigma Aldrich).

### Immunofluorescence microscopy image capture and quantitative expression analysis

Slides were immersed in Xylene for 2x5min to dewax and transferred to 100% Ethanol for 2x5min to rehydrate. Slides were immersed in 3% hydrogen peroxide in methanol for 20min and washed in Phosphate Buffered Saline (PBS) on a platform rocker for 5 min. Slides were blocked in 1% goat serum/0.1% BSA in PBS in a humidified chamber for 30 minutes at RT. Slides were incubated overnight at 4°C in primary antibody diluted in blocking serum (all 1:100 dilution). Slides were washed twice with PBS on a platform rocker for 5 minutes and incubated with AlexorFlour 488 labelled secondary antibody (all 1:500 in blocking serum) for 1h in the dark at RT. Slides were then washed 3x with PBS, mounted using DAPI Vectorshield (Vector, Peterborough, UK) and TIF images acquired on a Leica DM5000 B fluorescence microscope (Milton Keynes, UK). TIF images were uploaded directly to ImageJ and quantitative expression analysis performed as described in the instructions at https://imagej.nih.gov/ij/docs/guide/user-guide.pdf. The entire epithelium was assessed for FEP, dysplasia and surgical margins and representative areas captured (x400 magnification) for quantitative analysis. For T1 and T4 OSCC, where no or limited epithelium was present, the entire biopsy was assessed under low power (x100 magnification) for marker expression and representative areas from the surface to the deep invasive front of the tumour were captured for quantitative analysis. Expression of E-cadherin, EMP-1, 5T4, N-cadherin and CD44 were considered positive if localised at the cell surface (termed membranous expression (m)) or exhibited membrane and cytoplasmic (m/c) expression. Expression of markers was considered negative if localised within the cytoplasm, nucleus (n) or peri-nuclear regions of the cell or if no reactivity was observed (-ve). All samples were evaluated further by double blinded analysis of representative fluorescence images by three members of the Ward lab.

### Statistical analysis

Statistical analysis of individual marker expression within and between biopsies was performed using SPSS software except where stated below. Statistical significance was set at p<0.05. Coefficient of determination was calculated in Microsoft Excel by plotting the mean and SD of the IF values for each marker. Binominal z-value was calculated using SPSS by comparison with the clinical diagnosis for each biopsy. p-value of the binominal z-value was calculated using Graphpad (http://www.graphpad.com/quickcalcs/pvalue1.cfm), where p<0.05 represents statistically significant variation from the clinical diagnosis.

## Results

### Characterisation of antibodies recognising E-cadherin, EMP1, 5T4, N-cadherin and CD44 that are used in this study

**Fig 1:**
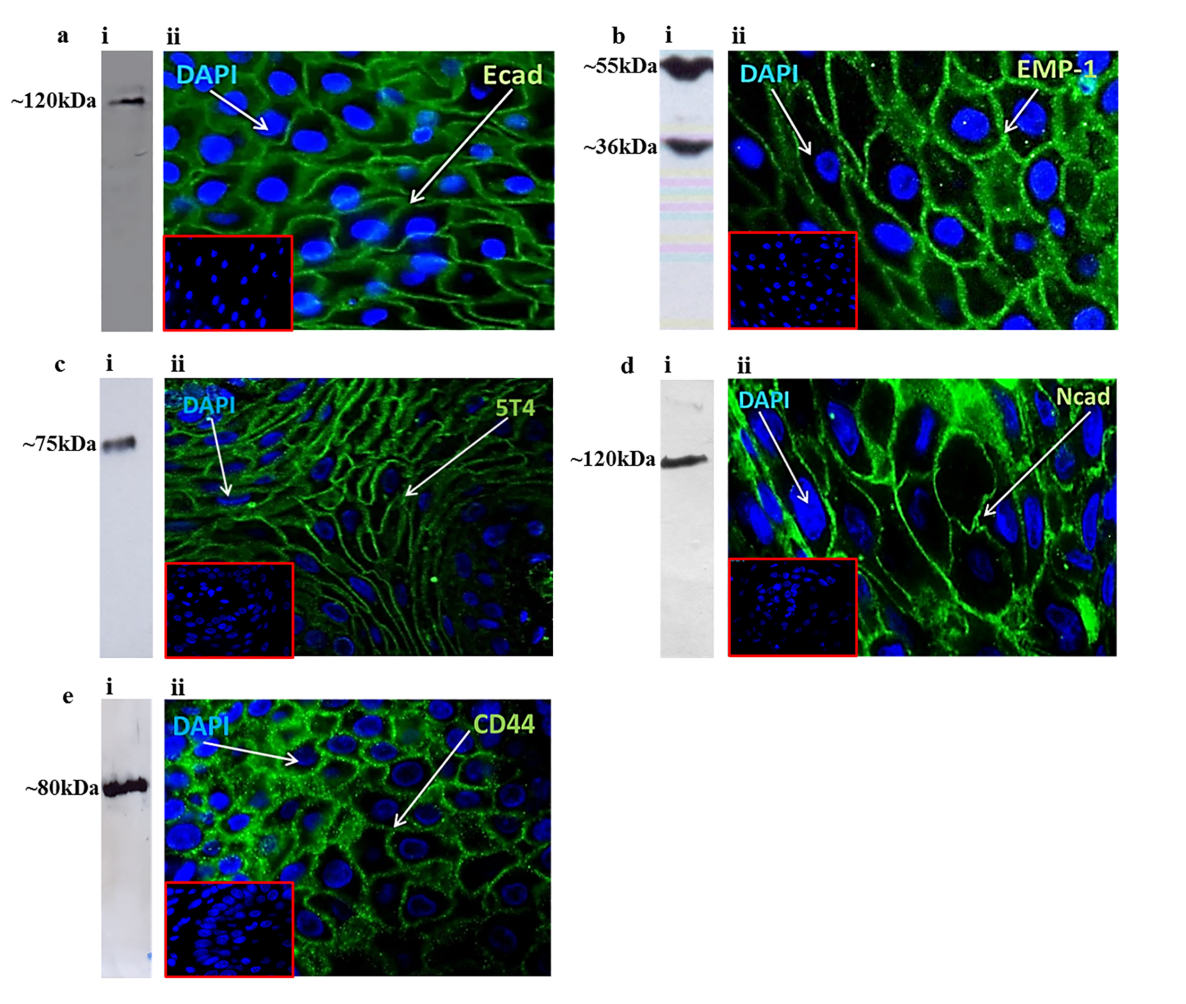
Characterisation of the antibodies used in this study. (a) E-cadherin, (b) EMP1, (c) 5T4, (d) N-cadherin and (e) CD44 expression assessed using (i) western blot and (ii) immunofluorescence microscopy analysis. Anti-E-cadherin antibody 610181 (BD Biosiences, UK) assessed using A549 cell lysates and normal oral epithelium biopsy; Anti-EMP1 antibody ab173224 (Abcam Plc, UK) assessed using MCF7 cell lysates and normal oral epithelium biopsy; Anti-5T4 antibody ab134162 (Abcam Plc, UK) was assessed using BicR56 cell lysates and normal oral epithelium biopsy; Anti-N-cadherin antibody ab76057 (Abcam Plc, UK) was assessed using A549 cell lysates and T4 OSCC biopsy; Anti-CD44 antibody ab40983 (Abcam Plc, UK) was assessed using MB-MDA-231 cell lysates and normal oral epithelium biopsy. Insets show appropriate negative control Ab staining. Green–antigen staining; Blue shows DAPI nuclear stain.

All antibodies employed in this study were assessed for reactivity by western blot analysis in appropriate cell lysates and confirmed for reactivity in paraffin-embedded biopsy sections prior to analysis (Fig 1). The anti-E-cadherin antibody 610181 (BD Biosciences) demonstrated reactivity with a single band of ~120kDa by western blot analysis in A549 total cell lysates (Fig 1A(i)) and provided clear evidence of plasma membrane localisation in a biopsy of healthy oral epithelium (NT; Fig 1A(ii)) using immunofluorescence microscopy. EMP1 expression was assessed using ab173224 (Abcam Plc) in MCF7 whole cell lysates using western blot analysis and showed bands at ~36kDa and ~55kDa (Fig 1B(i)). Multiple bands for EMP1 protein are common [25,26] and are often tissue dependent, potentially representing dimer/trimerization of the protein and/or altered glycosylation patterns. Cell surface localisation of EMP1 protein was evident in a biopsy of healthy epithelium using immunofluorescence microscopy analysis (Fig 1B(ii)). The 5T4 antigen was assessed using ab134162 (Abcam Plc) in western blot analysis of Bicr56 total cell lysates, showing a single band at ~75kDa (Fig 1C(i)). Plasma membrane localisation of 5T4 was evident using immunofluorescence microscopy analysis of a healthy oral epithelium biopsy (Fig 1C(ii)). The anti-N-cadherin antibody ab76057 (Abcam Plc) demonstrated reactivity with a single band of ~120kDa by western blot analysis in A549 total cell lysates (Fig 1D(i)) and exhibited plasma membrane localisation in a biopsy of T4 OSCC using immunofluorescence microscopy (Fig 1D(ii)). The anti-CD44 antibody ab40983 (Abcam, Plc) demonstrated reactivity with a single band of ~80kDa by western blot analysis in total lysates from MB-MDA-231 cells (Fig 1E(i)) and plasma membrane localisation in a biopsy of healthy oral epithelium using immunofluorescence microscopy analysis (Fig 1E(ii)). However, CD44 expression patterns did not reveal any statistical significance in any of the patient cohorts (Fisher’s exact test and one-way ANOVA analysis) and is not assessed further in this study. All data relating to CD44 expression in patient biopsies is contained within Table 1A and the Supporting Information section.

### Determination of cellular localisation and fluorescence intensity of marker expression in oral epithelium biopsies

**Fig 2:**
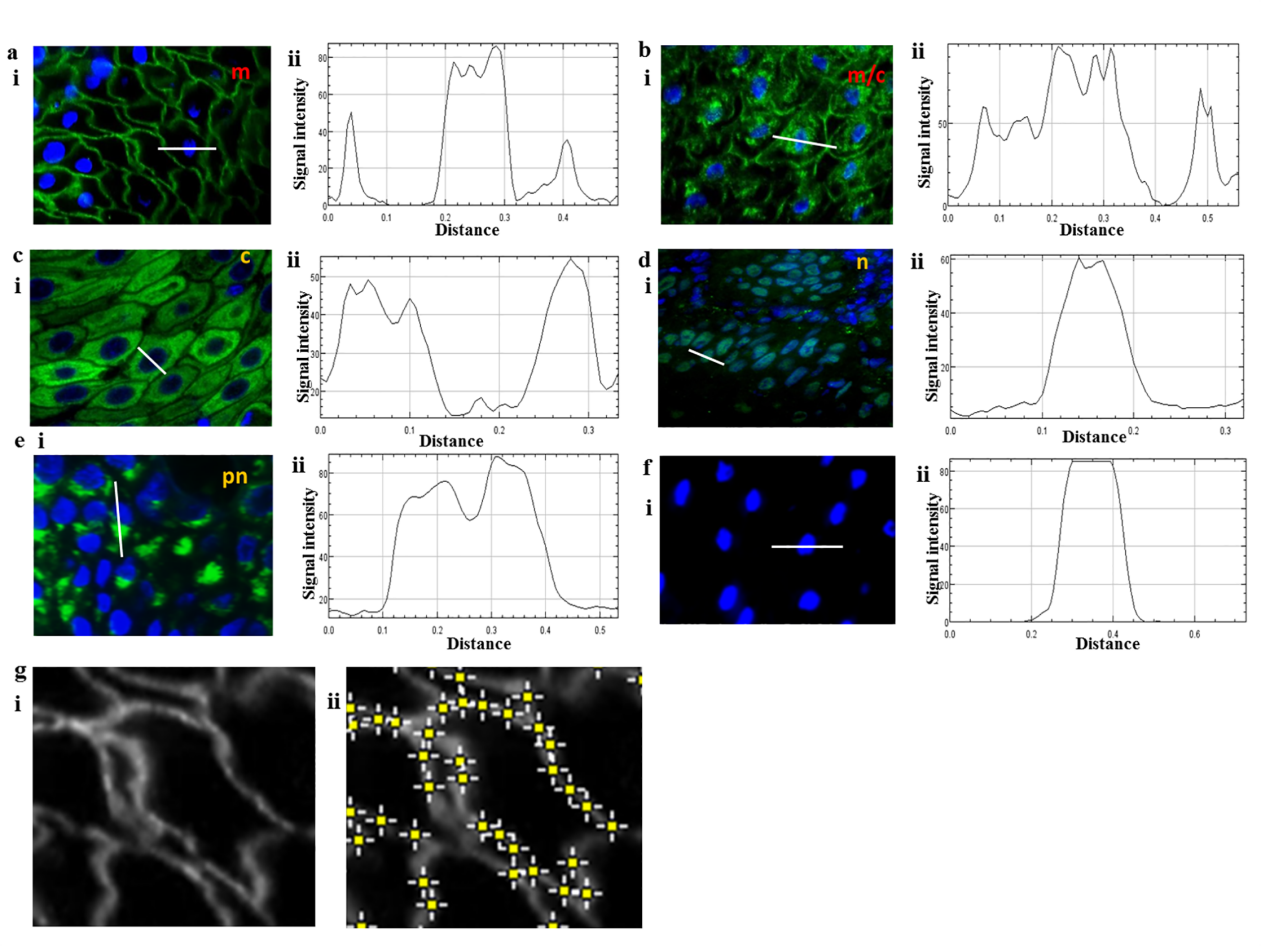
Determination of cellular localisation and fluorescence intensity of marker expression in oral epithelium biopsies. Cell surface localisation (a), cell surface and cytoplasmic localisation (b), cytoplasmic localisation (c), nuclear localisation (d), peri-nuclear localisation (e) and lack of expression (f) of individual protein markers was determined using (i) immunofluorescence microscopy analysis and (ii) cell localisation confirmed using the ‘Plot Profile’ application in ImageJ software. White lines on the images in (i) represent the region used for the Plot Profile analysis shown in (ii). (g) (i) Example of membrane localisation of marker expression in ImageJ software and (ii) quantification of fluorescence intensity using the ‘Find Maxima’ application in ImageJ software.

The expression of E-cadherin, EMP1, 5T4, N-cadherin and CD44 were demarcated into plasma membrane (Fig 2A; m), plasma membrane/cytoplasmic (Fig 2B; m/c), cytoplasmic (Fig 2C; c), nuclear (Fig 2D; n) and perinuclear (Fig 2E; pn) localisation. Protein localisation was confirmed using ImageJ analysis which allowed discrimination between the demarcations described above, demonstrating proof-of-principle for automated analysis of the expression and localisation of markers within biopsies (Fig 2A(ii)–2F(ii)). Fluorescence intensity of membrane staining was determined using ImageJ (Fig 2G) and mean intensity ±SD was recorded for each sample. Comparison of nuclear and peri-nuclear expression of the markers revealed no significant differences between NT, FEP, LGD, HGD, T1 OSCC and T4 OSCC and are not considered further (all p>0.05 ANOVA).

### Expression of E-cadherin, EMP1, 5T4 and N-cadherin in normal oral epithelium

E-cadherin was expressed at the plasma membrane in all normal oral epithelium biopsies (12/12; Table 1A), with 2/12 biopsies exhibiting both membrane and cytoplasmic expression (S1 Table). EMP1 and 5T4 were expressed at the plasma membrane in 11/12 biopsies (Table 1A), with most samples expressing plasma membrane expression only (S1 Table). N-cadherin plasma membrane reactivity was absent in 11/12 biopsies (Table 1A), with 7/12 samples exhibiting cytoplasmic localisation alone (S1 Table). E-cadherin, EMP1 and 5T4 exhibited statistically significant increased fluorescence levels compared to N-cadherin (Table 1B; p<0.001), therefore, normal oral epithelium is associated with plasma membrane expression of E-cadherin, EMP1 and 5T4 and absence of N-cadherin.

**Table 1:** Marker expression and fluorescence intensity in oral epithelium biopsies. (a) Cell surface expression of E-cadherin, EMP1, 5T4, N-cadherin and CD44 in the biopsies used in this study. (b) Fluorescence intensity readings from immunofluorescence microscopy analysis in NT, FEP, LGD, HGD, T1 OSCC and T4 OSCC and their expression within these biopsies (one-way ANOVA).

**A**										
**Diagnosis**	**CDH1**^**$**^		**EMP1**^**$**^		**5T4**^**$**^		**CDH2**^**$**^		**CD44**^**$**^	
	**+ve**	**-ve**	**+ve**	**-ve**	**+ve**	**-ve**	**+ve**	**-ve**	**+ve**	**-ve**
Normal Tissue	12	0	11	1	11	1	1	11	4	8
FEP	6	4	8	2	9	1	1	9	1	9
LGD	0	20	5	15	12	8	2	18	5	15
HGD	0	16	2	14	4	12	4	12	5	11
T1 OSCC	1	4	0	5	3	2	0	5	1	4
T4 OSCC	1	9	1	9	5	5	6	4	0	10
LGD-M	6	3	6	3	3	6	0	9	2	7
HGD-M	2	5	4	3	3	4	1	6	0	7
T1 OSCC-M	2	3	4	1	2	3	0	5	1	4
**B**										
**Diagnosis**	**CDH1** **Mean FI**^**|**^	**EMP1** **Mean FI**^**|**^	**5T4** **Mean FI**^**|**^	**CDH2** **Mean FI**^**|**^	CDH1 vs EMP1*	CDH1 vs 5T4*	CDH1 vs CDH2*	EMP1 vs 5T4*	EMP1 vs CDH2*	**5T4** **vs** CDH2*
**NT**	**53.1 (1.5/2.8)**	**55.5 (6.9/13.5)**	**42.9 (7.2/14.2)**	**6.3 (5.3/10.3)**	ns	ns	**0.0006**	ns	**0.0002**	**0.0003**
**FEP**	**30.4 (6.4/6.4)**	**45.7 (9.8/12.8)**	**46.2 (7.0/9.1)**	**4.6 (3.6/6.7)**	ns	ns	ns	ns	**0.017**	**0.004**
**LGD**	**0**	**11.2 (4.5/8.5)**	**31.3 (6.5/12.4)**	**2.8 (2.8/5.3)**	ns	**0.0001**	ns	**0.021**	ns	**0.001**
**HGD**	**0**	**7.4 (5.2/9.8)**	**12.2 (5.5/10.5)**	**11.6 (5.4/10.2)**	ns	ns	ns	ns	ns	ns
**T1 OSCC**	**8.7 (8.7/15.2)**	**0**	**26.0 (12.2/21.4)**	**0**	ns	ns	ns	ns	ns	ns
**T4 OSCC**	**6.5 (6.5/12.1)**	**4.4 (4.4/8.3)**	**30.3 (10.9/20.2)**	**37.1 (8.7/16.2)**	ns	ns	**0.041**	ns	**0.034**	ns

^$^Cell surface expression of markers shown.

^|^Standard error of the mean/Confidence Interval.

*One-Way ANOVA analysis (p-value); FI–relative fluorescence intensity; SEM–standard error of the mean; CI– 95% confidence interval; NT- normal tissue; FEP–fibroepithelial polyp; LGD–low grade dysplasia; HGD–high grade dysplasia; T1 OSCC–T1 stage oral squamous cell carcinoma; T4 OSCC–T4 stage OSCC. CDH1 –E-cadherin; EMP–EMP1; CDH2 –N-cadherin.

### Expression of E-cadherin, EMP1, 5T4 and N-cadherin in fibroepithelial polyps

Fibroepithelial polyp biopsies were originally intended for use as additional examples of normal oral epithelium. However, 4/10 biopsies lacked expression of E-cadherin (Table 1A) and 4/6 of the remaining biopsies exhibiting both cell surface and cytoplasmic localisation of the protein (S2 Table). Compared to NT biopsies, E-cadherin expression was significantly decreased in FEP samples and was associated with a large Hedges’ *g* effect size (Table 2A; p = 0.029, *g* 0.98). EMP1, 5T4 and N-cadherin expression was not statistically significant compared to normal epithelium (Table 2A). However, two-way ANOVA analysis showed a statistically significant difference in all marker expression between NT and FEP (Table 2A; p = 0.0037). Therefore, whilst FEP biopsies lacked any pathological features, the absence of E-cadherin in 40% of the biopsies suggests the presence of abnormality within the epithelium.

**Table 2:** Comparison of marker expression in abnormal tissue compared to normal epithelium. (a) Expression of E-cadherin, EMP1, 5T4 and N-cadherin in FEP, LGD, HGD, T1 OSCC and T4 OSCC biopsies compared to normal tissue (NT). (b) Expression of E-cadherin, EMP1, 5T4 and N-cadherin in FEP, LGD, HGD, T1 OSCC and T4 OSCC biopsies compared to an assumed linear relationship of FEP>LGD>HGD>T1 OSCC>T4 OSCC. (c) Hypothesis testing of an assumed linear NT>LGD>HGD relationship and putative linear HGD>T1 OSCC>T4 OSCC transition. Grey boxes show statistically significant results.

**A**					
**Diagnosis**	CDH1*† **(compared to NT)**	EMP1*† **(compared to NT)**	5T4*† **(compared to NT)**	CDH2*† **(compared to NT)**	All markers** (compared to NT)
**FEP**	**p = 0.029** **(*g* 0.98)**	p = 0.177 (*g* 0.32)	p = 0.998 (*g* 0.06)	p = 0.998 (*g* 0.06)	**p = 0.0037**
**LGD**	**p<0.00001** **(*g* >10)**	**p = 0.0006** **(*g* 1.74)**	p = 0.103 (***g* 1.03**)	p = 0.998 (*g* 0.14)	**p<0.0001**
**HGD**	**p<0.00001** **(*g* >10)**	**p = 0.00005** **(*g* 2.1)**	**p = 0.0006** **(*g* 1.76)**	p = 0.355 (*g* 0.44)	**p<0.0001**
**T1 OSCC**	**p = 0.0007** **(*g* 3.87)**	**p = 0.001** **(*g* 3.87)**	p = 0.191 **(*g* 1.12)**	p = 0.998 (*g* 0.34)	**p<0.0001**
**T4 OSCC**	**p = 0.00002** **(*g* 3.32)**	**p = 0.0003** **(*g* 3.04)**	p = 0.056 **(*g* 0.64)**	**p = 0.02** **(*g* 1.42)**	**p<0.0001**
**B**					
**Diagnosis**	CDH1*†	EMP1*†	5T4*†	CDH2*†	All markers**
**FEP vs LGD**	**p = 0.0004** **(*g* 1.65)**	**p = 0.007** **(*g* 1.3)**	p = 0.204 (*g* 0.69)	p = 0.998 (*g* 0.2)	**p<0.0001**
**LGD vs HGD**	p = 1 (*g* 0)	p = 0.410 (*g* 0.32)	**p = 0.049** **(*g* 0.75)**	p = 0.374 (*g* 0.61)	**p = 0.249**
**HGD vs T1 OSCC**	p = 0.273 (*g* 0.97)	p = 0.998 (*g* 0.43)	p = 0.28 (*g* 0.75)	p = 0.532 (*g* 0.65)	**p = 0.633**
**T1 OSCC vs T4 OSCC**	p = 0.542 (*g* 0.28)	p = 0.998 (*g* 0.4)	p = 0.998 (*g* 0.2)	**p = 0.04** **(*g* 1.84)**	**p = 0.179**
**C**					
**Hypothesis**		**CDH1**	**EMP1**	**5T4**	**CDH2**
**NT>LGD>HGD transition**		**r**^**2**^ **= 0.89** **F-value = 16.18** **p = 0.0027**	**r**^**2**^ **= 0.966** **F-value = 56.8** **p = 0.00008**	**r**^**2**^ **= 0.953** **F-value = 40.5** **p = 0.0002**	**r**^**2**^ **= 0.432** **F-value = 1.52** **p = 0.3**
**HGD>T1 OSCC>T4 OSCC transition**		**r**^**2**^ **= 0.25** **F-value = 0.67** **p = 0.603**	**r**^**2**^ **= 0.036** **F-value = 0.07** **p = 0.971**	**r**^**2**^ **= 0.48** **F-value = 1.85** **p = 0.239**	**r**^**2**^ **= 0.402** **F-value = 1.34** **p = 0.345**

*Fisher’s exact test (FET) of +ve/-ve expression

† Hedges’ *g* effect size analysis

**Two-Way ANOVA analysis (p value); NT- normal tissue; FEP–fibroepithelial polyp; LGD–low grade dysplasia; HGD–high grade dysplasia; T1 OSCC–T1 stage oral squamous cell carcinoma; T4 OSCC–T4 stage OSCC. CDH1 –E-cadherin; EMP–EMP1; CDH2 –N-cadherin. r^2^—coefficient of determination; F-value—Fisher F-value.

### Expression of E-cadherin, EMP1, 5T4 and N-cadherin in low-grade dysplasia

All low-grade dysplasia biopsies lacked plasma membrane expression of E-cadherin (Tables 1 and 2; S3 Table). EMP1 also exhibited statistically significant loss of plasma membrane staining in 15/20 biopsies (p = 0.006, FEP compared to NT; Tables 1 and 2). Whilst 5T4 expression was detected at the plasma membrane in 12/20 samples, this result was not significant when compared to NT (Tables 1 and 2). N-cadherin expression was detected in 2/20 biopsies which was not statistically significant compared to normal tissue (Tables 1 and 2). Two-way ANOVA analysis showed a statistically significant difference in all marker expression between NT and LGD (Table 2A; p<0.0001). Therefore, low-grade dysplasia biopsies lack plasma membrane expression of E-cadherin, exhibit statistically significant decreased EMP1 reactivity and do not exhibit any significant changes in 5T4 or N-cadherin expression.

### Expression of E-cadherin, EMP1, 5T4 and N-cadherin in high-grade dysplasia

All high-grade dysplasia biopsies lacked plasma membrane expression of E-cadherin (Tables 1 and 2; S4 Table; p = <0.000001, compared to NT; Table 2). EMP1 exhibited statistically significant loss of plasma membrane staining in 14/16 biopsies (p = 0.00005, compared to NT; Tables 1 and 2) and 5T4 was absent from the plasma membrane in 12/16 biopsies (p = 0.0006, compared to NT; Tables 1 and 2). N-cadherin plasma membrane expression was observed in 4/16 biopsies although this result was not statistically significant when compared to normal tissue (Tables 1 and 2). Two-way ANOVA analysis showed a statistically significant difference in all marker expression between NT and HGD (Table 2A; p<0.0001). Therefore, high-grade dysplasia biopsies lack plasma membrane expression of E-cadherin, exhibit statistically significant decreased EMP1 and 5T4 reactivity but do not exhibit any changes in N-cadherin protein expression compared to NT.

### Expression of E-cadherin, EMP1, 5T4 and N-cadherin in T1 OSCC

Only five T1 OSCC biopsies were assessed, therefore, some degree of caution is required in the interpretation of results from these samples. 4/5 biopsies lacked E-cadherin plasma membrane expression (Tables 1 and 2; S5 Table; p = 0.002, compared to NT) and all biopsies lacked plasma membrane localisation of EMP1 (Tables 1 and 2; S5 Table; p = 0.001, compared to NT). Plasma membrane localisation of 5T4 was detected in 3/5 biopsies (p>0.05, compared to NT) and N-cadherin was absent from all five biopsies (p>0.05, compared to NT). Two-way ANOVA analysis showed a statistically significant difference in all marker expression between NT and T1 OSCC (Table 2A; p<0.0001). Therefore, notwithstanding the small sample number, T1 OSCC biopsies lack plasma membrane expression of EMP1, N-cadherin and, mostly, E-cadherin and do not exhibit any statistically significant alterations in 5T4 or N-cadherin protein expression compared to NT.

### Expression of E-cadherin, EMP1, 5T4 and N-cadherin in T4 OSCC

9/10 T4 OSCC biopsies lacked E-cadherin and EMP1 plasma membrane expression (Tables 1 and 2, S6 Table; p = 0.00002 and 0.0003 respectively, compared to NT). Plasma membrane localisation of 5T4 was detected in 50% of the samples (p>0.05, compared to NT; Table 2B) and N-cadherin was detected at the plasma membrane of 6/10 biopsies (Tables 1 and 2; p = 0.02, compared to NT). Therefore, T4 OSCC biopsies lack plasma membrane expression of E-cadherin and EMP1 in 90% of biopsies and exhibit plasma membrane localisation of N-cadherin protein in 60% of cases. However, no statistically significant difference in 5T4 protein expression was observed compared to NT. Two-way ANOVA analysis showed a statistically significant difference in all marker expression between NT and T4 OSCC (Table 2A; p<0.0001).

In summary, compared to NT biopsies FEP is associated with lack of E-cadherin expression, LGD associated with loss of E-cadherin and EMP1 expression and HGD associated with lack of E-cadherin, EMP1 and 5T4 in the absence of gain of the EMT marker N-cadherin (Table 2A). This shows that loss of epithelial integrity is an early event associated with FEP and oral dysplasia and that this occurs in the absence of an E- to N-cadherin switch [27]. Compared to NT biopsies, T1 OSCC exhibits loss of E-cadherin and EMP1 and T4 OSCC is associated with absence of E-cadherin and EMP1 and gain of N-cadherin expression (Table 2A).

### Expression of E-cadherin, EMP1, 5T4 and N-cadherin between epithelial diseased states

Two-way ANOVA analysis of NT, FEP, LGD, HGD, T1 and T4 OSCC revealed E-cadherin loss alone is an early marker of NT>FEP transition, loss of E-cadherin and EMP1 an indicator of a putative FEP>LGD transition, loss of E-cadherin, EMP1 and 5T4 is indicative of LGD>HGD transition and gain of N-cadherin a marker of the T1>T4 OSCC transition (Table 2B). However, the combined marker expression could not discriminate between LGD>HGD, a putative HGD>T1 OSCC or T1>T4 OSCC transition (Table 2B; two-way ANOVA p>0.05). Therefore, the majority of the alterations in marker expression, except for N-cadherin, take place by the HGD stage of disease and are subsequently little altered.

It is generally accepted that OSCC arises as a linear transition from pre-cancerous lesions, such as HGD, however, the transition rate from dysplasia>OSCC ranges between 0.3 to 17.5%, suggesting some degree of uncertainty as to the exact mechanism of OSCC tumorigenesis. Noutomi et al (2006) [28] have shown that OSCC may arise from dysplasia via different genetic pathways, suggesting that multiple transition events may occur. Linear regression and coefficient of determination analysis was utilised to test the hypothesis of linear transition from HGD > T1 OSCC > T4 OSCC (Table 2C). The putative NT > LGD > HGD transition displayed positive correlation for E-cadherin (r^2^ = 0.89; p = 0.0027), EMP1 (r^2^ = 0.966; p = 0.00008) and 5T4 (r^2^ = 0.953; p = 0.0002) but not for N-cadherin (r^2^ = 0.432; p = 0.345). By contrast, the putative HGD > T1 OSCC > T4 OSCC linear transition was not statistically significant for any of the markers.

### Expression of E-cadherin, EMP1, 5T4 and N-cadherin in the safety margin of LGD, HGD and T1 OSCC biopsies

The expression of E-cadherin, EMP1, 5T4 and N-cadherin was assessed in the surgical margin of LGD, HGD and T1 OSCC biopsies that exhibited normal histology and a clear surgical safety margin (Table 3A and S7 Table). Of the 20 LGD, 16 HGD and 5 T1 OSCC biopsies, a total of 9, 7 and 5 biopsies exhibited histologically normal tissue within the surgical safety margin, respectively, and were used for further study. E-cadherin was expressed at the plasma membrane of 6/9 LGD margins (LGD-M; p = 0.063 compared to NT), 2/7 of HGD margins (HGD-M; p = 0.002) and 2/5 of T1 OSCC margins (T1-M; p = 0.015) (Table 3B). EMP1 and N-cadherin expression were not significantly different in any of the disease types compared to NT (Table 3B). 5T4 was significantly different in LGD and HGD margins compared to NT (Table 3B). However, comparison of all markers expressed in LGD-M, HGD-M and T1 OSCC-M revealed statistical significance for all samples compared to NT (Table 3B; two-way ANOVA).

**Table 3:** Marker expression in the surgical safety margin of LGD, HGD and T1 OSCC. (a) Cell surface expression of E-cadherin, EMP1, 5T4 and N-cadherin in the surgical safety margins of LGD, HGD and T1 OSCC biopsies. (b) Marker expression in LGD, HGD and T1 OSCC surgical safety margins compared to normal tissue. (c) Marker expression in LGD, HGD and T1 OSCC surgical safety margins compared to their respective biopsy samples. (d) Statistical significance of low-grade dysplasia (LGD), high-grade dysplasia (HGD) and T1 OSCC when using healthy oral epithelium (NT), fibroepithelial polyp or the respective surgical margins as a healthy control. Grey boxes show statistically significant results.

**A**						
**Diagnosis**	**CDH1** **Mean FI (SEM/CI)**	**EMP1** **Mean FI (SEM/CI)**	**5T4** **Mean FI (SEM/CI)**	**CDH2** **Mean FI (SEM/CI)**		
**LGD-M**	**33.6 (9.2/17.9)**	**34.5 (11.3/22.2)**	**22.9 (11.5/22.5)**	**0**		
**HGD-M**	**17.0 (11.8/21.4)**	**18.1 (7.7/14.1)**	**27.2 (13.7/24.9)**	**8.6 (8.6/15.6)**		
**T1 OSCC-M**	**29.7 (18.6/32.6)**	**39.1 (11.2/19.7)**	**21.5 (13.2/23.1)**	**0**		
**B**						
**Diagnosis**	CDH1* (compared to NT)	EMP1* (compared to NT)	5T4* (compared to NT)	CDH2* (compared to NT)	All markers compared to NT**	
**LGD-M**	p = 0.063	p = 0.272	**p = 0.016**	p = 0.998	**p = 0.001**	
**HGD-M**	**p = 0.002**	p = 0.117	**p = 0.038**	p = 0.438	**p<0.0001**	
**T1 OSCC-M**	**p = 0.015**	p = 0.515	p = 0.053	p = 0.998	**p = 0.0002**	
**C**						
**Diagnosis**	CDH1*compared to non-margin	EMP1*compared to non-margin	5T4*compared to non-margin	CDH2*compared to non-margin	All markers compared to non-margin**	
**LGD-M**	**p = 0.0002**	p = 0.272	p = 0.245	p = 0.998	**p = 0.013**	
**HGD-M**	p = 0.142	**p = 0.045**	p = 0.625	p = 0.998	**p = 0.029**	
**T1 OSCC-M**	p = 0.538	**p = 0.048**	p = 0.998	p = 0.998	**p = 0.127**	

**D**									
**Diagnosis**	CDH1*			EMP1*			5T4*		
	**Margin**	**FEP**	**NT**	**Margin**	**FEP**	**NT**	**Margin**	**FEP**	**NT**
**LGD**	**p = 0.0001**	**p = 0.0004**	**p = 0.00001**	**p = 0.047**	**p = 0.007**	**p = 0.0006**	p = 0.245	p = 0.203	p = 0.103
**HGD**	p = 0.083	**p = 0.0009**	**p = 0.00001**	**p = 0.045**	**p = 0.001**	**p = 0.00005**	p = 0.625	**p = 0.004**	**p = 0.0006**
**T1 OSCC**	p = 0.538	p = 0.282	**p = 0.01**	**p = 0.048**	**p = 0.007**	**p = 0.001**	p = 0.998	p = 0.242	p = 0.191

**FI–relative fluorescence intensity; SEM–standard error of the mean; CI– 95% confidence interval; LGD-M = low grade dysplasia safety margin; HGD-M = high grade dysplasia; safety margin; T1 OSCC-M = T1 stage oral squamous cell carcinoma safety margin. CDH1 –E-cadherin; EMP–EMP1; CDH2 –N-cadherin.**

*Fisher’s exact test (FET) of +ve/-ve expression;

**Two-Way ANOVA analysis (p value); LGD = low grade dysplasia; HGD = high grade dysplasia; T1 OSCC = T1 stage oral squamous cell carcinoma. CDH1 –E-cadherin; EMP–EMP1; CDH2 –N-cadherin.

Comparison of the surgical margin marker expression with their associated LGD, HGD or T1 OSCC biopsies showed statistically significant difference in E-cadherin expression in LGD vs LGD-M and decreased EMP1 expression in HGD vs HGD-M and T1 OSCC vs T1 OSCC-M (Table 3C). This shows that utilisation of safety margins as a representation of normal oral epithelium underestimates the statistical significance in all samples and introduces Type II errors for E-cadherin in HGD and T1 OSCC and for 5T4 in HGD (Table 3D). Similarly, utilisation of FEP biopsies as a positive epithelial control also underestimates statistical significance in all samples and introduces a Type II error for E-cadherin expression in T1 OSCC (Table 3D).

### Prediction of abnormal epithelium and disease grades using marker expression in FEP, LGD, HGD, T1 and T4 OSCC biopsies

Based on the statistical analysis of marker expression, normal epithelium was classified as Ecad^+^/EMP^+^/5T4^+^/Ncad^-^, abnormal epithelium (e.g. FEP) as Ecad^-^/EMP^+^/5T4^+^/Ncad^-^, LGD as Ecad^-^/EMP^-^/5T4^+^/Ncad^-^, HGD as Ecad^-^/EMP^-^/5T4^-^/Ncad^-^, T1 OSCC as Ecad^-^/EMP^-^/5T4^+/-^/Ncad^-^ and T4 OSCC as Ecad^-^/EMP^-^/5T4^+/-^/Ncad^+^ (Table 4). Comparison of these classifications to marker expression in each individual biopsy predicted 16% of normal epithelium (z-value = 1.48), 50% of FEP (z = 2.58) and 100% of LGD, HGD, T1 and T4 OSCC (all z = 0) biopsies to be abnormal (S1, S2, S3, S4, S5 and S6 Tables). Using these criteria, we also assessed each biopsy for prediction of disease stage (S1, S2, S3, S4, S5 and S6 Tables). 60% of FEP biopsies (z = 2.58), 80% of LGD biopsies (z = 2.1), 63% of HGD biopsies (z = 2.43), 40% of T1 OSCC (z = 2.07) and 70% of T4 OSCC (z = 1.87) could be classified. Removal of 5T4 from the classification criteria described in Table 4 resulted in 8% of NT samples being classed as abnormal (z = 1.02) and maintained 100% prediction of abnormal epithelium in LGD, HGD, T1 OSCC and T4 OSCC biopsies. In addition, these criteria improved the classification scores of LGD (z = 1.47), HGD (z = 2.14), T1 OSCC (z = 1.05) and T4 OSCC (z = 1.49) biopsies (S1, S2, S3, S4, S5 and S6 Tables). The margins of LGD, HGD and T1 OSCC were also assessed for abnormality using the marker classification shown in Table 4. 89% of LGD-M (z = 3.79), 86% of HGD-M (z = 3.24) and 80% of T1 OSCC-M (z = 2.52) biopsies were classified as abnormal (S7 Table), showing significant difference between the pathological analysis and marker expression data.

**Table 4:** Summary of marker expression in healthy, abnormal and diseased epithelium. Marker expression used to classify normal tissue (NT), fibroepithelial polyp (FEP), low-grade dysplasia (LGD), high-grade dysplasia (HGD), T1 OSCC and T4 OSCC.

Classification*	CDH1	EMP1	5T4	CDH2
**NT**	**+ve**	**+ve**	**+ve**	**-ve**
**FEP**	**+ve/-ve**	**+ve**	**+ve**	**-ve**
**LGD**	**-ve**	**-ve**	**+ve**	**-ve**
**HGD**	**-ve**	**-ve**	**-ve**	**-ve**
**T1 OSCC**	**-ve**	**-ve**	**+ve /-ve**	**-ve**
**T4 OSCC**	**-ve**	**-ve**	**+ve /-ve**	**+ve**

*NT–normal tissue; A–abnormal tissue; LGD–low grade dysplasia; HGD–high grade dysplasia; T1 OSCC–T1 stage oral squamous cell carcinoma; T4 OSCC–T4 stage oral squamous cell carcinoma. CDH1 –E-cadherin; EMP–EMP1; CDH2 –N-cadherin

## Discussion

OSCC is the most common cancer in the oral cavity and is associated with poorer 5-year survival rate compared to many other cancers. The disease is further compounded by the difficulty in predicting pre-cancerous abnormal tissues that will progress to OSCC and the high recurrence rates following surgical resection of the latter. At present, the mechanism of transition from normal tissue to dysplasia/OSCC is elusive and transformation rates of between 0.3 to 17.5% for dysplasia to OSCC [1] suggests significant heterogeneity between patient populations. Therefore, methods that can detect abnormal tissue within the oral cavity prior to OSCC formation will allow the biological and epidemiological aspects of this disease to be further studied and may aid elucidation of the molecular mechanisms associated with OSCC tumorigenesis.

Our study shows that a panel of markers can be utilised to assess the epithelial state of oral tissues, comprising of E-cadherin, EMP1, 5T4 and N-cadherin. E-cadherin represented the most sensitive of these markers, in terms of high expression in normal tissue, and was absent in early stages associated with abnormal epithelium. Previous studies have demonstrated mixed results using loss of E-cadherin expression as an early marker of abnormal epithelium [18,19]. In this study we have utilised an antibody recognising the intracellular domain of E-cadherin as this region functions in both structural actin binding and cellular signalling. Furthermore, the extracellular domain of E-cadherin is liable to cleavage by MMPs resulting in a soluble 80kDa fragment that can subsequently bind to cellular E-cadherin protein, disrupting signalling and epithelial integrity. As shown, the cytoplasmic E-cadherin epitope is rapidly lost from dysplastic tissue, and a significant proportion of FEP biopsies, and therefore represents a sensitive method for detecting abnormality within epithelial tissue biopsies. In addition, it demonstrates that loss of E-cadherin is an early event occurring in abnormal oral epithelium. We suggest that the mixed results in other studies for E-cadherin marker expression represents the use of uncharacterised antibodies and/or ones that recognise epitopes within the extracellular domain of E-cadherin.

Interestingly, an E- to N-cadherin switch was not observed in any of the dysplastic or T1 OSCC biopsies that lacked E-cadherin expression. An E- to N-cadherin switch is associated with increased motility and invasion of epithelial cells during embryo development and tumour cell metastasis [24]. Elevated N-cadherin expression was only observed in T4 stage OSCC biopsies, which is consistent with the study of Zhou et al (2015) [27], where Vimentin expression was associated only with metastatic OSCC. This demonstrates that EMT is a late event associated with OSCC and that loss of E-cadherin alone is insufficient to induce EMT *in vivo*, as we have previously reported in embryonic stem cells [20]. These results support our previously published DENT hypothesis for the role of E-cadherin in early tumorigenesis [23]. A non-linear trend was observed for a putative transition from HGD>T1 OSCC> T4 OSCC whilst NT>LGD>HGD exhibited a statistically significant linear progression. These results suggest that the putative HGD>T1 OSCC transition may not occur, that the T1 OSCC derives from a subpopulation of dysplastic cells or that re-epithelialization is required for transition from HGD to a cancerous state.

Utilisation of E-cadherin, EMP1 and N-cadherin marker expression allowed prediction of abnormal epithelium in all pathologically abnormal biopsies. In addition, diseased states could be predicted in at least 75% of samples. Therefore, use of E-cadherin, EMP1 and N-cadherin may provide a useful means to predict abnormal and diseased epithelial states in biopsies, and the addition of further markers to this panel may allow increased discrimination and predictive capabilities. However, further analysis will be required to confirm this classification in unrelated oral dysplasia and SCC biopsies. This method also predicted abnormal epithelium in the margins of LGD, HGD and T1 OSCC, which may account for the high recurrence rate associated with this disease. Statistical analysis of marker expression in diseased tissue compared to the ‘healthy’ safety margin underestimated the statistical significance in all samples and introduced Type II errors. Furthermore, utilisation of FEP biopsies as a ‘healthy’ control also underestimated statistical significance in all samples and introduced a Type II error for 5T4 expression in HGD. Therefore, tissue biopsies used as healthy control samples in the comparison of diseased biopsies can have a marked impact upon statistical analysis and should be assessed for the presence of healthy epithelium prior to use.

## Supporting information

S1 Table Localisation and expression of E-cadherin, EMP1, 5T4, N-cadherin and CD44 in normal tissue biopsies. ‘Classification’ column shows the predicted state of the biopsy (normal tissue–NT; abnormal–A) using the marker classification table shown in Table 4. ‘Disease prediction’ columns show the predicted diseased grade of the biopsy using the marker classification table shown in Table 4 (Ecad/EMP/5T4/Ncad) or using E-cadherin, EMP1 and N-cadherin (Ecad/EMP/Ncad). ‘A’ is shown where no grade prediction was possible. Z-value and corresponding p-value is shown for each column prediction compared to the clinical diagnosis (i.e. all NT).
(TIF)

S2 Table Localisation and expression of E-cadherin, EMP1, 5T4, N-cadherin and CD44 in fibroepithelial polyp biopsies. ‘Classification’ column shows the predicted state of the biopsy (normal tissue–NT; abnormal–A) using the marker classification table shown in Table 4. ‘Disease prediction’ columns show the predicted diseased grade of the biopsy using the marker classification table shown in Table 4 (Ecad/EMP/5T4/Ncad) or using E-cadherin, EMP1 and N-cadherin (Ecad/EMP/Ncad). ‘A’ is shown where no grade prediction was possible. Z-value and corresponding p-value is shown for each column prediction compared to the clinical diagnosis.
(TIF)

S3 Table Localisation and expression of E-cadherin, EMP1, 5T4, N-cadherin and CD44 in low-grade dysplasia biopsies. ‘Classification’ column shows the predicted state of the biopsy (normal tissue–NT; abnormal–A) using the marker classification table shown in Table 4. ‘Disease prediction’ columns show the predicted diseased grade of the biopsy using the marker classification table shown in Table 4 (Ecad/EMP/5T4/Ncad) or using E-cadherin, EMP1 and N-cadherin (Ecad/EMP/Ncad). ‘A’ is shown where no grade prediction was possible. Z-value and corresponding p-value is shown for each column prediction compared to the clinical diagnosis.
(TIF)

S4 Table Localisation and expression of E-cadherin, EMP1, 5T4, N-cadherin and CD44 in high-grade dysplasia biopsies. ‘Classification’ column shows the predicted state of the biopsy (normal tissue–NT; abnormal–A) using the marker classification table shown in Table 4. ‘Disease prediction’ columns show the predicted diseased grade of the biopsy using the marker classification table shown in Table 4 (Ecad/EMP/5T4/Ncad) or using E-cadherin, EMP1 and N-cadherin (Ecad/EMP/Ncad). ‘A’ is shown where no prediction was possible. Z-value is shown for each column prediction. ‘A’ is shown where no grade prediction was possible. Z-value and corresponding p-value is shown for each column prediction compared to the clinical diagnosis.
(TIF)

S5 Table Localisation and expression of E-cadherin, EMP1, 5T4, N-cadherin and CD44 in T1 OSCC biopsies. ‘Classification’ column shows the predicted state of the biopsy (normal tissue–NT; abnormal–A) using the marker classification table shown in Table 4. ‘Disease prediction’ columns show the predicted diseased grade of the biopsy using the marker classification table shown in Table 4 (Ecad/EMP/5T4/Ncad) or using E-cadherin, EMP1 and N-cadherin (Ecad/EMP/Ncad). ‘A’ is shown where no grade prediction was possible. Z-value and corresponding p-value is shown for each column prediction compared to the clinical diagnosis.
(TIF)

S6 Table Localisation and expression of E-cadherin, EMP1, 5T4, N-cadherin and CD44 in T4 OSCC biopsies. ‘Classification’ column shows the predicted state of the biopsy (normal tissue–NT; abnormal–A) using the marker classification table shown in Table 4. ‘Disease prediction’ columns show the predicted diseased grade of the biopsy using the marker classification table shown in Table 4 (Ecad/EMP/5T4/Ncad) or using E-cadherin, EMP1 and N-cadherin (Ecad/EMP/Ncad). ‘A’ is shown where no grade prediction was possible. Z-value and corresponding p-value is shown for each column prediction compared to the clinical diagnosis.
(TIF)

S7 Table Localisation and expression of E-cadherin, EMP1, 5T4, N-cadherin and CD44 in the surgical margin of low-grade dysplasia, high-grade dysplasia and T1 OSCC biopsies. ‘Classification’ column shows the predicted state of the biopsy (normal tissue–NT; abnormal–A) using the marker classification table shown in Table 4. ‘Disease prediction’ columns show the predicted diseased grade of the biopsy using the marker classification table shown in Table 4 (Ecad/EMP/5T4/Ncad) or using E-cadherin, EMP1 and N-cadherin (Ecad/EMP/Ncad). ‘A’ is shown where no prediction was possible. Z-value and corresponding p-value is shown for each column prediction compared to the clinical diagnosis (i.e. all NT).
(TIF)
