# Supplementary figures and images for: Loss of epithelial markers is an early event in oral dysplasia and is observed within the safety margin of dysplastic and T1 OSCC biopsies
Source: PLoS One. 2017 Dec 7;12(12):e0187449. doi: 10.1371/journal.pone.0187449 (PMC5720771; doi:10.1371/journal.pone.0187449)

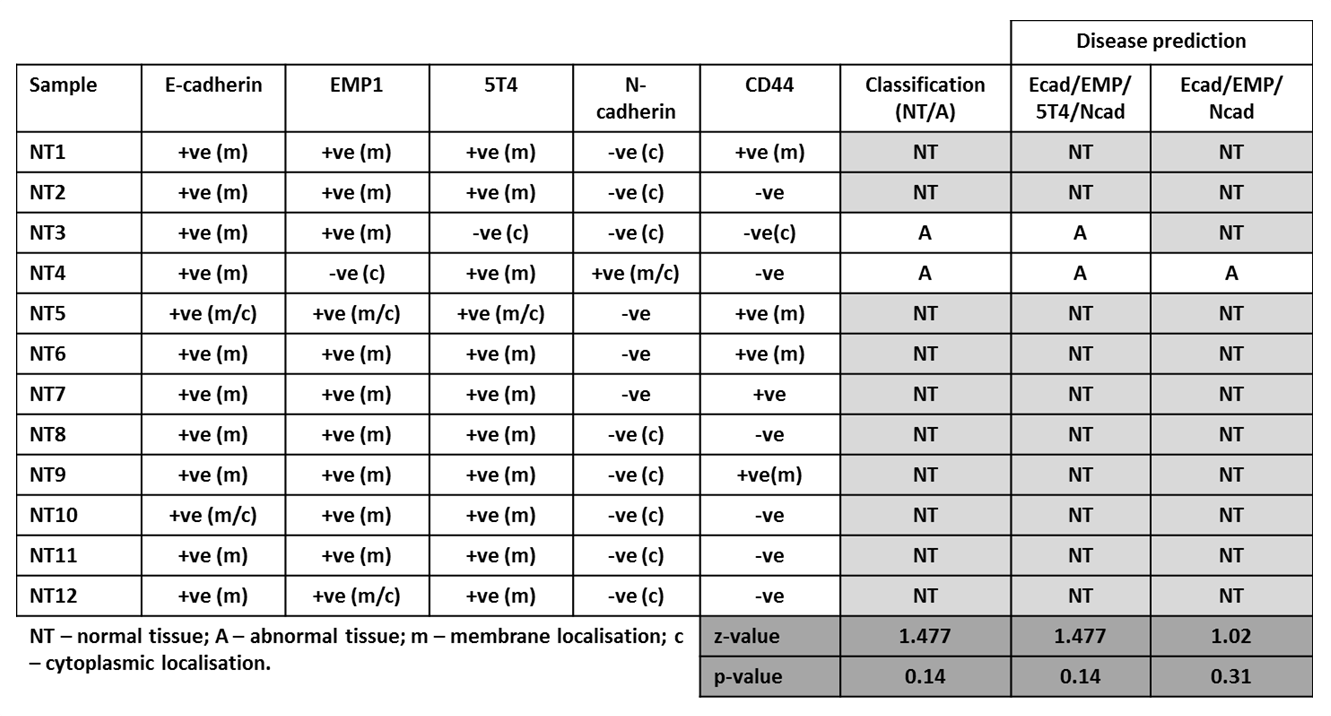

Supplement: S1 Table — ‘Classification’ column shows the predicted state of the biopsy (normal tissue–NT; abnormal–A) using the marker classification table shown in Table 4. ‘Disease prediction’ columns show the predicted diseased grade of the biopsy using the marker classification table shown in Table 4 (Ecad/EMP/5T4/Ncad) or using E-cadherin, EMP1 and N-cadherin (Ecad/EMP/Ncad). ‘A’ is shown where no grade prediction was possible. Z-value and corresponding p-value is shown for each column prediction compared to the clinical diagnosis (i.e. all NT). (TIF) [file pone.0187449.s001.tif]

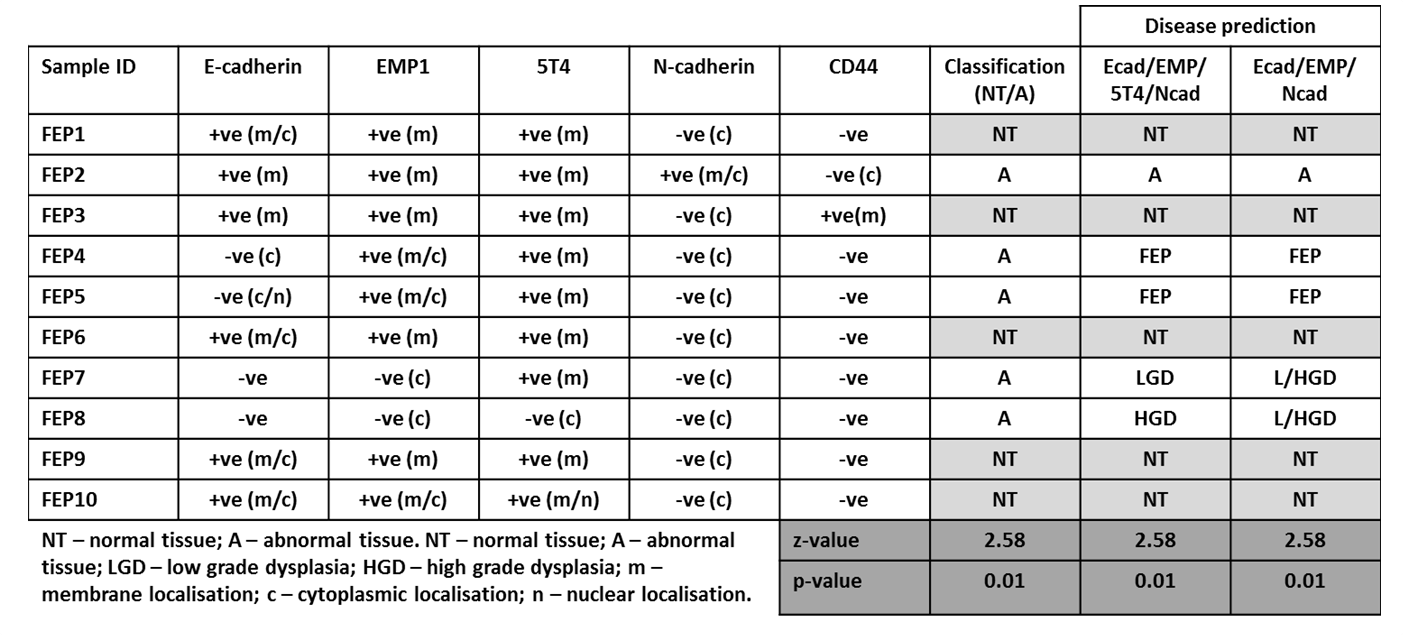

Supplement: S2 Table — ‘Classification’ column shows the predicted state of the biopsy (normal tissue–NT; abnormal–A) using the marker classification table shown in Table 4. ‘Disease prediction’ columns show the predicted diseased grade of the biopsy using the marker classification table shown in Table 4 (Ecad/EMP/5T4/Ncad) or using E-cadherin, EMP1 and N-cadherin (Ecad/EMP/Ncad). ‘A’ is shown where no grade prediction was possible. Z-value and corresponding p-value is shown for each column prediction compared to the clinical diagnosis. (TIF) [file pone.0187449.s002.tif]

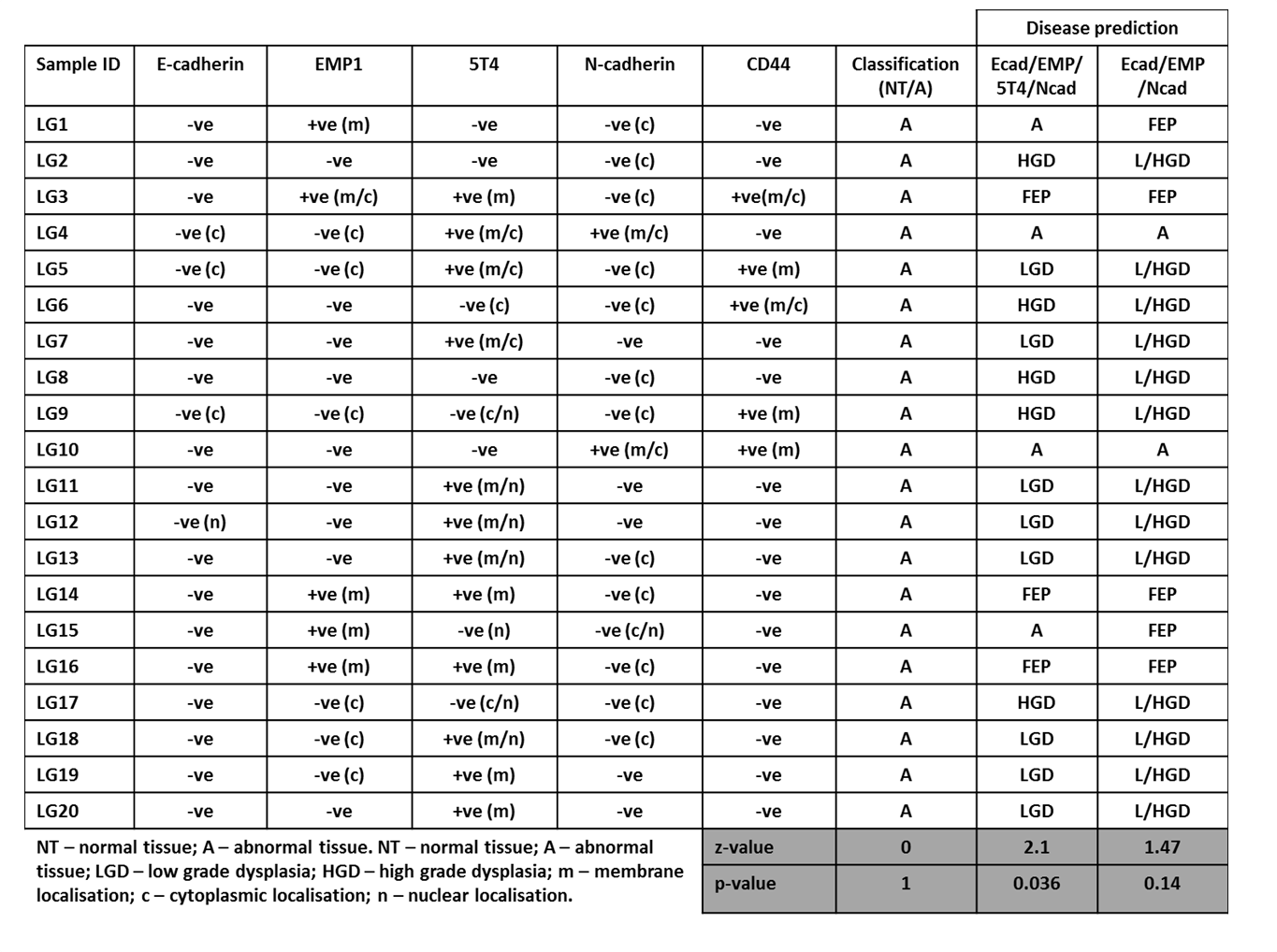

Supplement: S3 Table — ‘Classification’ column shows the predicted state of the biopsy (normal tissue–NT; abnormal–A) using the marker classification table shown in Table 4. ‘Disease prediction’ columns show the predicted diseased grade of the biopsy using the marker classification table shown in Table 4 (Ecad/EMP/5T4/Ncad) or using E-cadherin, EMP1 and N-cadherin (Ecad/EMP/Ncad). ‘A’ is shown where no grade prediction was possible. Z-value and corresponding p-value is shown for each column prediction compared to the clinical diagnosis. (TIF) [file pone.0187449.s003.tif]

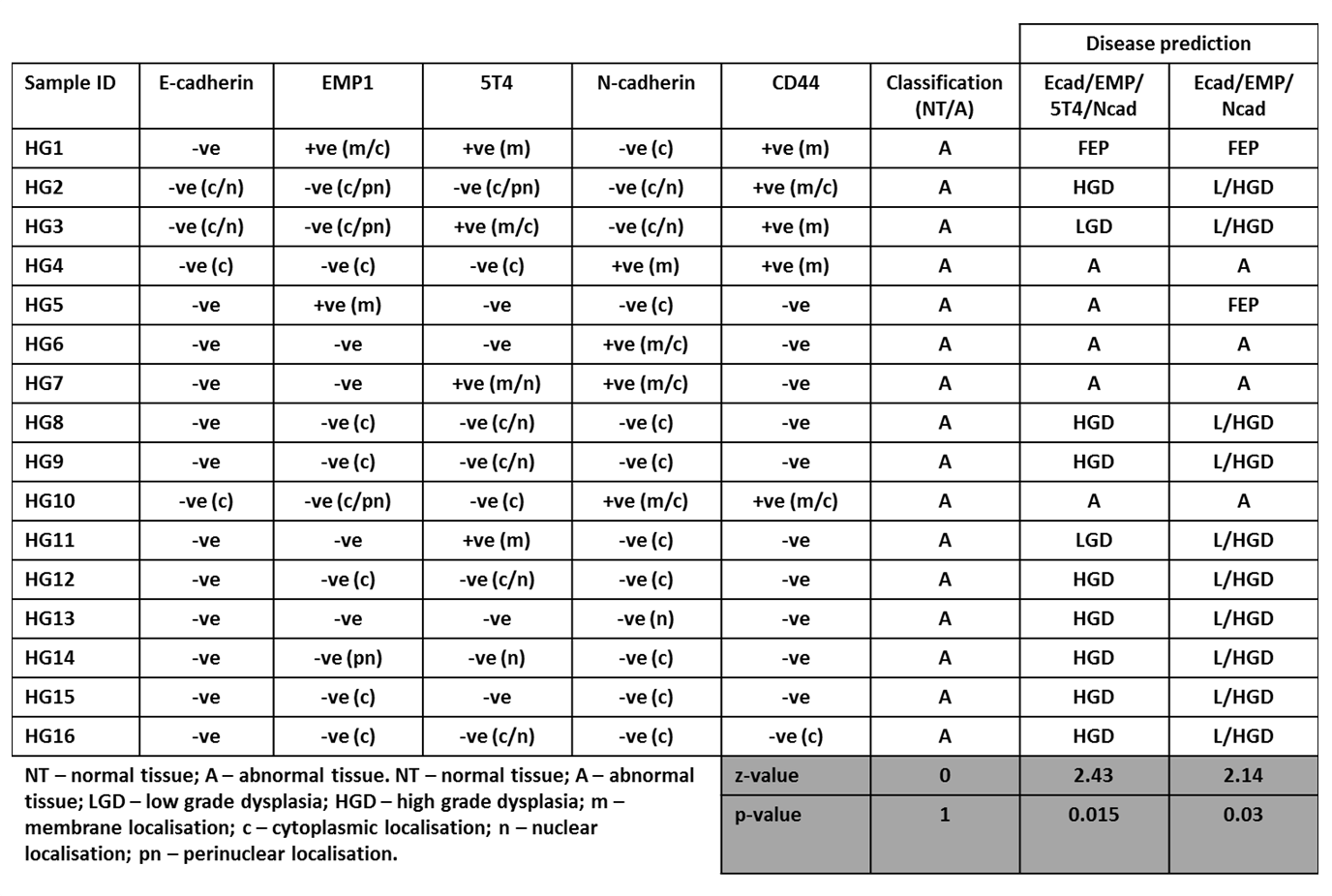

Supplement: S4 Table — ‘Classification’ column shows the predicted state of the biopsy (normal tissue–NT; abnormal–A) using the marker classification table shown in Table 4. ‘Disease prediction’ columns show the predicted diseased grade of the biopsy using the marker classification table shown in Table 4 (Ecad/EMP/5T4/Ncad) or using E-cadherin, EMP1 and N-cadherin (Ecad/EMP/Ncad). ‘A’ is shown where no prediction was possible. Z-value is shown for each column prediction. ‘A’ is shown where no grade prediction was possible. Z-value and corresponding p-value is shown for each column prediction compared to the clinical diagnosis. (TIF) [file pone.0187449.s004.tif]

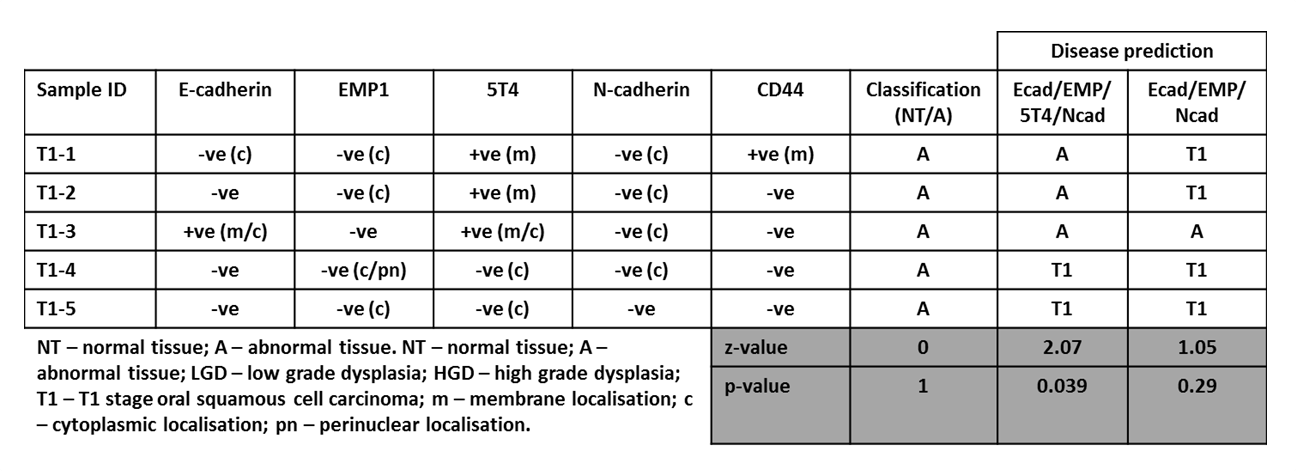

Supplement: S5 Table — ‘Classification’ column shows the predicted state of the biopsy (normal tissue–NT; abnormal–A) using the marker classification table shown in Table 4. ‘Disease prediction’ columns show the predicted diseased grade of the biopsy using the marker classification table shown in Table 4 (Ecad/EMP/5T4/Ncad) or using E-cadherin, EMP1 and N-cadherin (Ecad/EMP/Ncad). ‘A’ is shown where no grade prediction was possible. Z-value and corresponding p-value is shown for each column prediction compared to the clinical diagnosis. (TIF) [file pone.0187449.s005.tif]

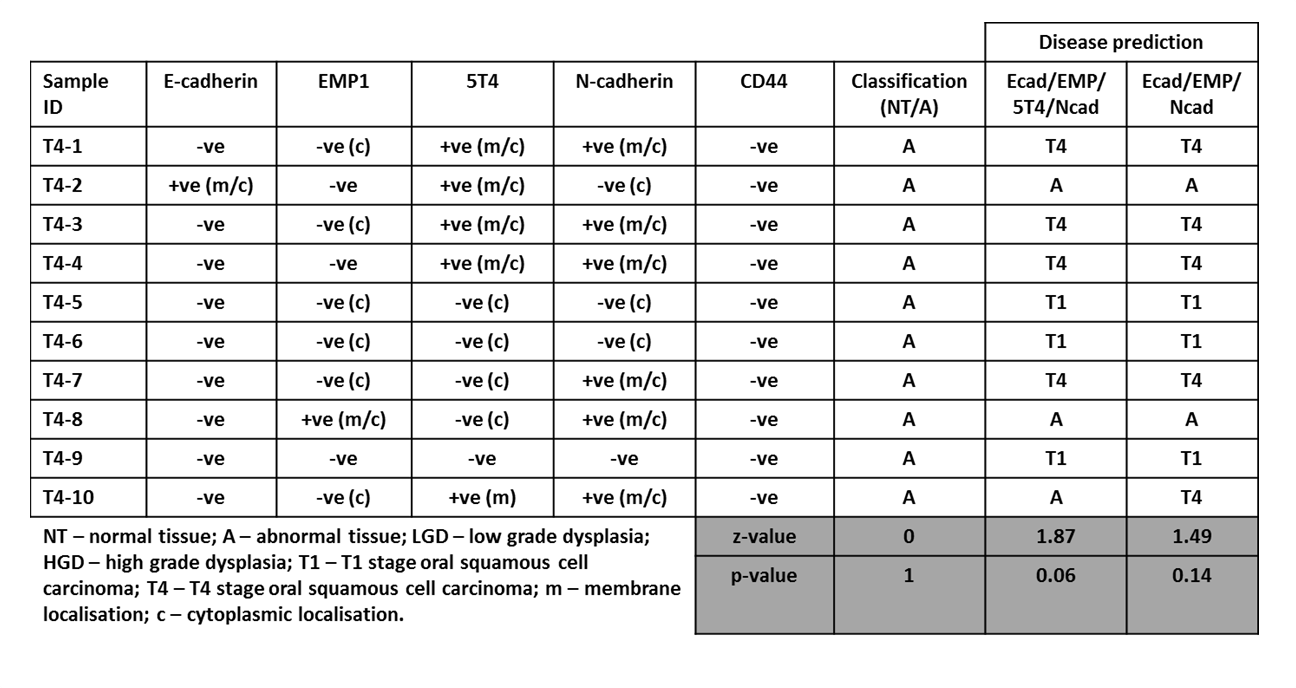

Supplement: S6 Table — ‘Classification’ column shows the predicted state of the biopsy (normal tissue–NT; abnormal–A) using the marker classification table shown in Table 4. ‘Disease prediction’ columns show the predicted diseased grade of the biopsy using the marker classification table shown in Table 4 (Ecad/EMP/5T4/Ncad) or using E-cadherin, EMP1 and N-cadherin (Ecad/EMP/Ncad). ‘A’ is shown where no grade prediction was possible. Z-value and corresponding p-value is shown for each column prediction compared to the clinical diagnosis. (TIF) [file pone.0187449.s006.tif]

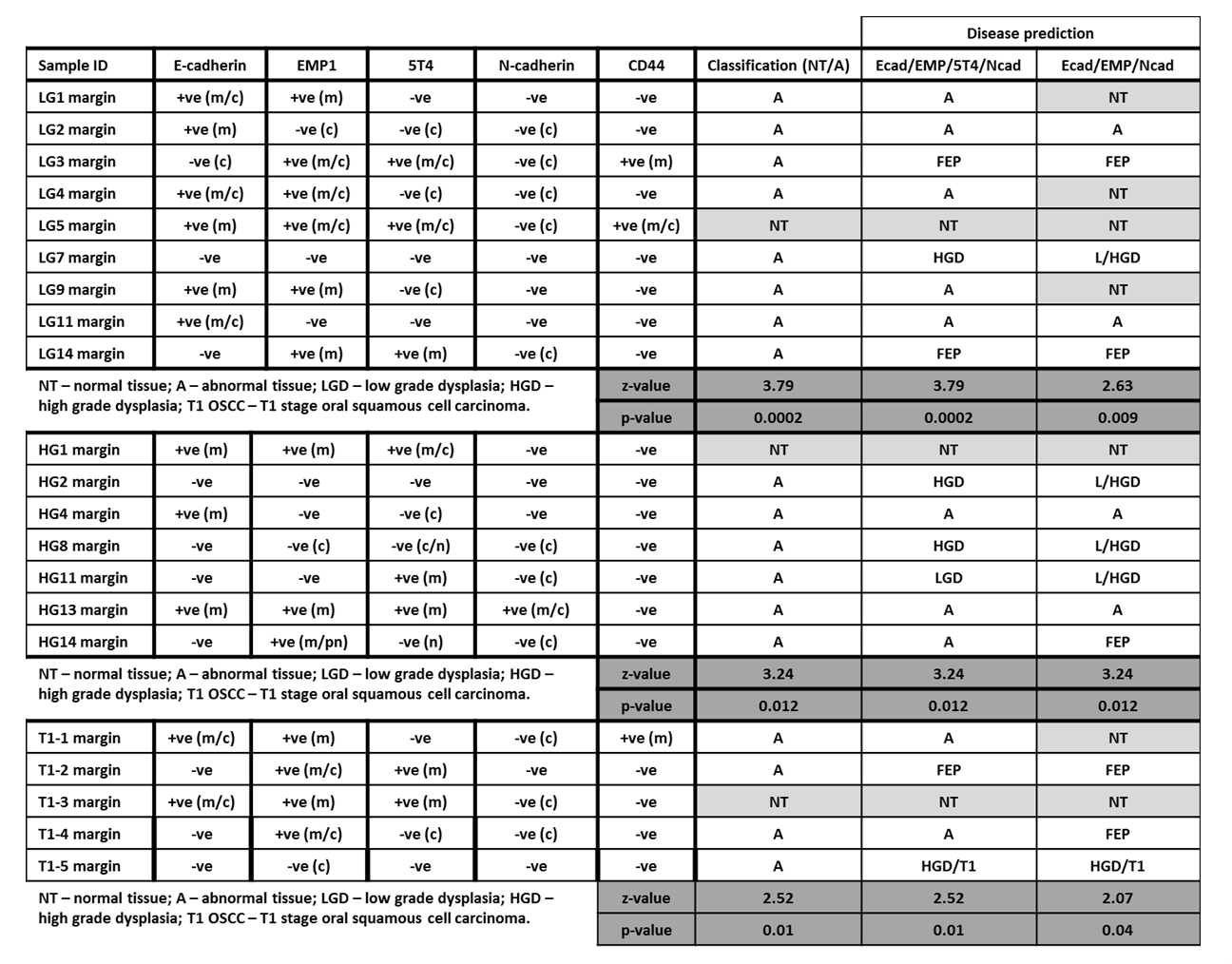

Supplement: S7 Table — ‘Classification’ column shows the predicted state of the biopsy (normal tissue–NT; abnormal–A) using the marker classification table shown in Table 4. ‘Disease prediction’ columns show the predicted diseased grade of the biopsy using the marker classification table shown in Table 4 (Ecad/EMP/5T4/Ncad) or using E-cadherin, EMP1 and N-cadherin (Ecad/EMP/Ncad). ‘A’ is shown where no prediction was possible. Z-value and corresponding p-value is shown for each column prediction compared to the clinical diagnosis (i.e. all NT). (TIF) [file pone.0187449.s007.tif]
